# Increment of plasma glucose by exogenous glucagon is associated with present and future renal function in type 2 diabetes:a retrospective study from glucagon stimulation test

**DOI:** 10.1186/s12902-019-0428-6

**Published:** 2019-10-15

**Authors:** Yasutaka Takeda, Yukihiro Fujita, Ryoichi Bessho, Mao Sato, Tomoe Abe, Tsuyoshi Yanagimachi, Hidemitsu Sakagami, Atsuko Abiko, Yumi Takiyama, Tsuguhito Ota, Masakazu Haneda

**Affiliations:** 1 Division of Metabolism and Biosystemic Science, Department of Internal Medicine, Asahikawa Medical University, 2-1-1-1 Midorigaoka Higashi, Asahikawa, 078-8510 Japan; 2 Division of Diabetology, Endocrinology and Nephrology, Department of Internal Medicine, Shiga University of Medical Science, Otsu, Japan

**Keywords:** Glucagon, ΔGlucose, Free fatty acid, Renal function, Gluconeogenesis

## Abstract

**Background:**

Glucagon stimulation test (GST) is often employed to assess the insulin reserve of the pancreatic beta cells in diabetic subjects. The clinical significance of the increment of plasma glucose (Δglucose) by exogenous glucagon during GST has not been elucidated. We investigated the relationship between Δglucose and clinical parameters including the liver and renal function in type 2 diabetic subjects, since we hypothesized that Δglucose is associated with the liver and renal function reflecting the capacity for gluconeogenesis in the organs.

**Methods:**

A total of 209 subjects with type 2 diabetes who underwent GST during admission were included in this cross-sectional study. We defined the difference between plasma glucose at fasting and 6 min after intravenous injection of 1 mg glucagon as Δglucose. We assessed correlations between Δglucose and clinical parameters such as diabetic duration, BMI, HbA1c, beta cell function, serum free fatty acids (FFA) which is known to stimulate gluconeogenesis, liver function, the indices of liver function, renal function, and urinary albumin excretion (UAE).

**Results:**

In correlation analysis, Δglucose positively correlated to FFA and estimated glomerular filtration rate (eGFR), but inversely to serum creatinine and cystatin C, although Δglucose showed no correlation with both liver function and the indices of residual liver function. Multiple regression analysis revealed that Δglucose was an independent determinant for the eGFR after 1 year, equally BMI, HbA1c, serum lipids, and UAE, which are known as the predictors for the development of chronic kidney disease.

**Conclusion:**

Our results suggest that Δglucose during GST might be related to gluconeogenesis in the kidney and could be the determinant of future renal function in type 2 diabetes.

## Background

Glucagon, secreted from pancreatic alpha cells, promotes hepatic glucose output (HGO) by stimulating both gluconeogenesis and glycogenolysis in the liver to maintain blood glucose levels and also stimulates insulin secretion from islet beta cells in glucose-independent manner in humans [1]. Glucagon stimulation test (GST) has been often used to assess the insulin reserve of the pancreatic beta cells in subjects with diabetes by measuring plasma C-peptide immunoreactivity (CPR) levels before and 6 min after exogenous intravenous glucagon injection [2, 3]. Although the increment of plasma glucose levels (Δglucose) by exogenous glucagon during GST is presumed to reflect a part of glucose output from the liver that might be attributed to gluconeogenesis and glycogenolysis, the clinical significance of Δglucose has not been elucidated.

The kidney also plays crucial roles in glucose metabolism via gluconeogenesis, glucose utilization, glucose reabsorption and glucose disposal from the renal glomerular filtrate. After an overnight fast, 50% of the glucose release into the circulation is the result of glycogenolysis in the liver and the other half is due to gluconeogenesis from lactate, glycerol, alanine and other amino acids both in liver and kidney [4]. Focusing on the gluconeogenesis, the kidney provides almost equal amounts of glucose to the liver into the circulation in the post-absorptive state [4].

In addition to insulin resistance in the liver, hyperglucagonemia in both fasting and postprandial states leads to increased rates of HGO, a major factor in the elevation of the blood glucose levels in type 2 diabetes mellitus (T2DM) [1]. On the other hand, Meyer and colleagues unveiled that renal glucose release as a result of gluconeogenesis into the circulation is increased in subjects with T2DM and its increment is comparable to the liver [5]. Consequently, glucose overproduction probably resulting from increased gluconeogenesis from both the liver and the kidney is closely involved in hyperglycemia in T2DM.

Indeed, gluconeogenesis can be modified in pathophysiological condition in the liver and the kidney. In 1970s, Frizzell and colleagues reported that spontaneous hypoglycemia in patients with renal failure occurred in association with decreased renal glucose production [6]. Furthermore, Garber et al. showed that an inadequate delivery of alanine, that is an important gluconeogenic substrate in the kidney, can cause fasting hypoglycemia in renal failure [7]. On the other hand, a recent study using magnetic resonance spectroscopy revealed that gluconeogenesis in patients with severe cirrhosis was decreased [8]. Interestingly, Joseph and colleagues revealed that extrahepatic tissues, especially the kidney, make a significant contribution to increased endogenous glucose production by gluconeogenesis in cases during anhepatic phase of liver transplantation without glucose production from the liver [9]. Thus, the liver and the kidney functions are thought to be related to gluconeogenesis in each organ.

Consequently, we hypothesized that Δglucose by exogenous glucagon during GST can reflect gluconeogenesis in not only the liver but also the kidney, and associate with the liver and renal function in T2DM. To validate this hypothesis, we investigated the relationship in subjects with T2DM between Δglucose and clinical parameters such as aspartate aminotransferase (AST) and alanine aminotransferase (ALT) as liver function, serum creatinine, cystatin C and estimated glomerular filtration rate (eGFR) as renal function, serum free fatty acids (FFA) which is known to stimulate gluconeogenesis, and albumin, cholinesterase (ChE), bilirubin, total cholesterol, triglyceride, high-density lipoprotein (HDL) cholesterol, low-density lipoprotein (LDL) cholesterol, and prothrombin time (PT) employed as the indices reflecting residual liver function.

## Methods

### Participants

We included a total of 209 (108 male and 101 female) Japanese patients with T2DM who underwent GST during admission in Asahikawa Medical University Hospital from 2013 to 2016, in this cross-sectional study. There was no participant who had symptomatic cerebrovascular disorders, severe heart disease, hepatitis and liver cirrhosis, renal failure (exclusion criteria: eGFR below 30 ml min^− 1^ 1.73 m^− 2^ or serum creatinine above 2.0 mg/dl), and malignant neoplasm, who was in the pre- or post-operative period, or who was positive for diabetes-related autoantibodies. The protocol of the current study was approved by the ethics committee of Asahikawa Medical University (Approval number: 17104).

### Study protocol and measurement

Prior to GST, all inpatient participants were treated with appropriate diet and insulin-based glucose lowering treatments after administration, resulting in fasting glucose levels and pre-meal glucose levels of 140 mg/dl or less to avoid glucotoxicity. No subjects received rapid-acting insulin after 6 pm on the 1 day before GST. Some participants received long acting insulin such as insulin glargine, insulin detemir or insulin degludec subcutaneously at 8 pm the day before GST to maintain adequate fasting blood glucose levels. Then, GST was carried out at 8 am after an overnight fast by measuring serum CPR at fasting and 6 min after single intravenous injection of 1 mg glucagon (Novo Nordisk, Tokyo, Japan). We defined the increment of serum CPR after glucagon injection as ΔCPR, the difference between plasma glucose at fasting and 6 min after glucagon injection as Δglucose. Blood and urine samples for laboratory measurements were collected at overnight fast. Height and body weight (BW) were measured, and body mass index (BMI) was calculated by dividing BW (kg) by height squared (m^2^). Plasma glucose levels were measured by automatic analyzer (GA09, A&T, Fujisawa, Japan) using glucose oxidase method. HbA1c levels were measured by the automatic analyzer (HLC-723G8, TOSOH Bioscience, Tokyo, Japan) using high performance liquid chromatography (HPLC) method. Both plasma insulin concentrations as immunoreactive insulin (IRI) and serum CPR were measured using an electrochemiluminescence method (cobas 6000, Roche Diagnostics Japan, Tokyo, Japan). Other biochemical values were measured by the automatic analyzer (LABOSPECT 008, HITACHI High-Technologies, Tokyo, Japan).

We assessed correlations between Δglucose and clinical parameters including BMI, duration of diabetes, HbA1c, serum CPR during GST, urinary CPR, serum lipids, serum FFA, liver function, the indices of residual liver function, renal function, and urinary albumin excretion (UAE). As renal function, we employed serum creatinine, cystatin C and eGFR. We calculated eGFR using the previously established equation for eGFR in Japanese subjects, which was calculated from serum creatinine, gender, and age in individual subjects [10].

### Statistical analysis

Data are expressed as mean ± SEM. The correlation coefficient was determined using Pearson’s correlation coefficient. We performed a multiple linear regression analysis to evaluate clinical parameters independently showing significant correlations with eGFR 1 year later among 119 participants whom we could continuously follow up after the discharge. Data were analyzed using GraphPad Prism 5 (GraphPad Software Inc., San Diego, CA, USA) and Ekuseru-Toukei 2015 (Social Survey Research Information Co., Ltd., Tokyo, Japan). A *p*-value < 0.05 was considered statistically significant.

## Results

### Clinical characteristics and parameters

The clinical characteristics and parameters of the total 209 participants are shown in Table 1. The mean age was 60.7 ± 1.0 years, mean BMI was 27.5 ± 0.4 kg/m^2^, mean diabetic duration was 13.0 ± 0.8 years, and mean HbA1c level was 9.7 ± 0.1% at the time of administration. For the GST, the mean plasma glucose at fasting and 6 min after glucagon injection were 122.2 ± 1.6 and 140.4 ± 1.6 mg/dl, respectively, and the mean Δglucose was 18.2 ± 0.5 mg/dl. Additionally, there was no significant difference in Δglucose among subjects with treated by glucose lowering agents, especially with or without biguanides or dipeptidyl peptidase-4 (DPP-4) inhibitors, which may influence the value of Δglucose (data not shown).

**Table 1:** Clinical characteristics and parameters

	*n* = 209		
Gender (male/female)	108 / 101		
Age (years)	60.7	±	1.0
BMI (kg/m^2^)	27.5	±	0.4
Systolic blood pressure (mmHg)	116.6	±	0.9
Diastolic blood pressure (mmHg)	67.6	±	0.7
Diabetic duration (years)	13.0	±	0.8
HbA1c (%)	9.7	±	0.1
Glucose at 0 min (mg/dl)	122.2	±	1.6
Glucose at 6 min (mg/dl)	140.4	±	1.6
ΔGlucose (mg/dl)	18.2	±	0.5
Serum CPR at 0 min (ng/ml)	1.84	±	0.08
Serum CPR at 6 min (ng/ml)	3.56	±	0.14
ΔCPR (ng/ml)	1.72	±	0.07
AST (IU/l)	31.6	±	1.8
ALT (IU/l)	35.9	±	2.6
Albumin (g/dl)	3.97	±	0.03
Cholinesterase (IU/l)	340.4	±	5.5
Total Bilirubin (mg/dl)	0.80	±	0.02
PT-INR	0.99	±	0.01
Total Cholesterol (mg/dl)	186.5	±	2.7
Triglyceride (mg/dl)	146.1	±	6.3
HDL-Cholesterol (mg/dl)	44.1	±	0.9
LDL-Cholesterol (mg/dl)	116.7	±	2.3
Apolipoprotein A^−1^ (mg/dl)	124.1	±	1.5
Apolipoprotein B (mg/dl)	95.9	±	1.5
Apolipoprotein E (mg/dl)	4.5	±	0.1
Lipoprotein (a) (mg/dl)	20.7	±	1.7
Free Fatty Acid (mEq/l)	0.56	±	0.02
Remnant Like Particle Cholesterol (mg/dl)	5.37	±	0.35
Serum Creatinine (mg/dl)	0.76	±	0.02
Estimated GFR (ml/min/1.73m^2^)	82.4	±	2.3
≧30 and < 45, n (%)		24	(11.5)
≧45 and < 60, n (%)		23	(11.0)
≧60 and < 90, n (%)		86	(41.1)
≧90, n (%)		76	(36.4)
Serum Cystatin C (mg/dl)	0.90	±	0.02
Urinary Albumin (mg/gCr)	257.6	±	55.7
Urinary Albumin (mg/day)	256.0	±	50.0
Treatment			
Glucose lowering agents			
Insulin, n (%)		162	(77.5)
Biguanides, n (%)		59	(28.2)
Sulfonylureas, n (%)		27	(12.9)
DPP-4 inhibitors, n (%)		118	(56.4)
GLP-1 R agonists, n (%)		17	(8.1)
Others, n (%)		43	(20.6)
Hypertension, n (%)		109	(52.2)
Antihypertensive agents in subjects with hypertension			
Angiotensin-converting enzyme inhibitors, n (%)		7	(6.4)
Angiotensin receptor blockers, n (%)		79	(72.5)
Calcium channel blockers, n (%)		72	(66.1)
Diuretics, n (%)		33	(30.3)
Alpha-blockers, n (%)		10	(9.2)
Beta-blockers, n (%)		5	(4.6)

The data are presented as means ± SEM. *BMI* Body mass index, *CPR* C-peptide immunoreactivity, *AST* Aspartate aminotransferase, *ALT* Alanine aminotransferase, *PT-INR* Prothrombin time-international normalized ratio, *HDL* High-density lipoprotein, *LDL* Low-density lipoprotein, *GFR* Glomerular filtration rate, *DPP-4* Dipeptidyl peptidase-4, *GLP-1* Glucagon-like peptide-1

### Correlations between Δglucose and clinical parameters

**Fig. 1:**
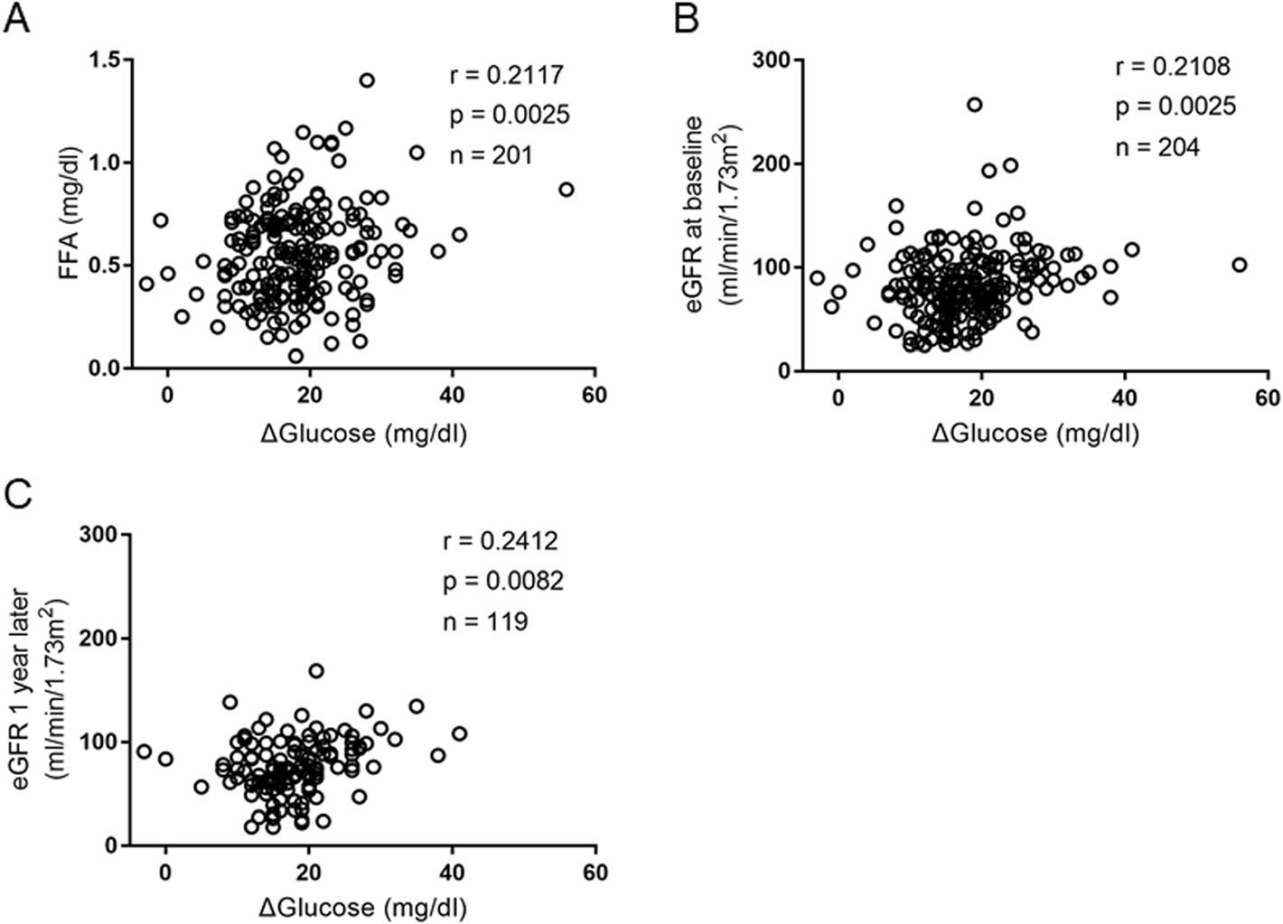
Correlation between Δglucose and FFA, eGFR at baseline, and eGFR 1 year later. Pearson’s correlation coefficients between Δglucose and free fatty acids (**a**), eGFR at baseline (**b**), and eGFR one year later (**c**)

First, we examined the correlations between Δglucose and clinical parameters as follows: age, BMI, duration of diabetes, HbA1c, serum CPR during GST, daily urinary CPR, serum lipids, FFA, liver function and residual liver function, renal function, and UAE (Table 2). We observed positive correlation between Δglucose and FFA (*r* = 0.2117, *p* = 0.0025; Fig. 1a), which is known to stimulate gluconeogenesis. Interestingly, Δglucose showed positive correlation with eGFR at baseline (*r* = 0.2108, *p* = 0.0025; Fig. 1b), and inverse correlation with serum creatinine (*r* = − 0.2166, *p* = 0.0017) and cystatin C (*r* = − 0.1515, *p* = 0.0302). On the other hand, we observed no relationship between Δglucose and liver function such as AST and ALT, and the indices of residual liver function such as serum albumin, ChE, bilirubin, PT, total cholesterol, triglyceride, HDL cholesterol, and LDL cholesterol.

**Table 2:** Correlation between Δglucose and clinical parameters

Variables		r	*p* value
Age (years)	–	0.0631	0.3641
BMI (kg/m^2^)		0.0261	0.7071
Diabetic duration (years)	–	0.1238	0.0741
HbA1c (%)	–	0.1165	0.0929
Serum CPR at 0 min (ng/ml)	–	0.1069	0.1235
Serum CPR at 6 min (ng/ml)	–	0.0352	0.6124
ΔCPR (ng/ml)		0.0572	0.4108
Urinary CPR (μg/day)	–	0.0282	0.6875
AST (IU/l)	–	0.0659	0.343
ALT (IU/l)	–	0.0777	0.2634
Albumin (g/dl)		0.0073	0.9162
Cholinesterase (IU/l)		0.0332	0.6354
Total Bilirubin (mg/dl)		0.0555	0.4252
PT-INR	–	0.1315	0.0602
Total Cholesterol (mg/dl)	–	0.0087	0.9007
Triglyceride (mg/dl)		0.1092	0.1155
HDL-Cholesterol (mg/dl)		0.0215	0.7569
LDL-Cholesterol (mg/dl)	–	0.0523	0.4518
Apolipoprotein A^−1^ (mg/dl)		0.1029	0.145
Apolipoprotein B (mg/dl)		0.03093	0.6621
Apolipoprotein E (mg/dl)		0.03105	0.6609
Lipoprotein (a) (mg/dl)		0.03749	0.5972
Free Fatty Acid (mg/dl)		0.2117	0.0025
Remnant Like Particle Cholesterol (mg/dl)		0.1065	0.1322
Serum Creatinine (mg/dl)	–	0.2166	0.0017
Estimated GFR (ml/min/1.73m^2^)		0.2108	0.0025
Serum Cystatin C (mg/dl)	–	0.1515	0.0302
Urinary Albumin (mg/gCr)		0.005094	0.9418
Urinary Albumin (mg/day)	–	0.01433	0.838

Pearson’s correlation coeficients between Δglucose and clinical parameters

### Determinants for the future renal function in multiple regression analysis

Since we observed that Δglucose correlated with present renal function, we hypothesized that Δglucose is also related to the future renal function and could be a determinant of the future renal function. Then, we further performed multiple regression analysis to identify the variables associated with eGFR after 1 year. We observed positive relationship between Δglucose and eGFR 1 year later (*r* = 0.2412, *p* = 0.0082; Fig. 1c) similarly to eGFR at baseline. Intriguingly, multiple regression analysis revealed that BMI (β = 0.1830, *p* = 0.0337), HbA1c (β = 0.2909, *p* = 0.0008), total cholesterol (β = − 1.4550, *p* = 0.0119), HDL-cholesterol (β = 0.5937, *p* = 0.0053), LDL-cholesterol (β = 1.3343, *p* = 0.0073), UAE (β = − 0.3141, *p* = 0.0040), and Δglucose (β = 0.2308, *p* = 0.0034) were independent determinants of the eGFR after 1 year (Table 3).

**Table 3:** Determinants of eGFR one year later in multiple linear regression analysis

Factors		β	*p* value
BMI		0.1830	0.0337
Diabetic duration	–	0.0377	0.6462
HbA1c		0.2909	0.0008
Systolic blood pressure	–	0.0272	0.7669
Diastolic blood pressure		0.0459	0.6151
ΔGlucose		0.2308	0.0034
ΔCPR		0.1048	0.2296
AST	–	0.0280	0.8451
ALT		0.0171	0.9080
Albumin	–	0.0597	0.5522
Total Cholesterol	–	1.4550	0.0119
Triglyceride		0.1628	0.3667
HDL-Cholesterol		0.5937	0.0053
LDL-Cholesterol		1.3343	0.0073
Free Fatty Acid		0.0889	0.2808
Urinary Albumin (mg/day)	–	0.3141	0.0040

Multiple regression analysis. Response variable, eGFR one year later

## Discussion

The clinical significance of Δglucose as the increment of plasma glucose levels during GST has not yet been elucidated, although it is assumed mostly to reflect a part of HGO by glucagon. To the best of our knowledge, this is the first study to investigate the relationship between Δglucose during GST and various clinical parameters in subjects with T2DM.

First, we demonstrated that Δglucose was positively associated with FFA. FFA is known to increase HGO via stimulation of gluconeogenesis in the liver. The mechanisms how FFA stimulates gluconeogenesis can be attributed to the generation of 1) acetyl-CoA derived from FFA oxidation, which activates pyruvate carboxylase, 2) nicotinamide adenine dinucleotide (NADH), which is used for the formation of glyceraldehyde 3-phosphate from 1,3-bisphosphoglycerate, and 3) adenosine triphosphate (ATP), which is used as an energy source [11]. Chen and colleague showed that increment of plasma FFA levels results in increased gluconeogenesis, and FFA positively correlates to gluconeogenesis in humans [12]. Based on these previous findings, the positive relationship between Δglucose by exogenous glucagon and FFA in this study is assumed to reflect the increment of blood glucose levels by FFA-associated gluconeogenesis in the liver.

Second, we anticipated that Δglucose was associated with both liver function or the indices of residual liver function, since the liver is the most important organ supplying glucose into the circulation in the body and Δglucose is presumably to reflect a part of HGO [1]. However, we observed no relationship between Δglucose and liver function such as AST and ALT, or the indices of residual liver function such as serum albumin, ChE, bilirubin, PT, and cholesterol. Why was Δglucose not associated with both liver function or the indices of residual liver function? Indeed, AST and ALT are established as the clinical indices of liver function, but these parameters are valuable only when serum levels are elevated at the liver injury or inflammation. In other words, we presume that both AST and ALT might not be perfect indicators which directly reflect the functional capacity of the liver. Additionally, the liver function of the participants in the current study was almost within normal range and was in a relatively narrow range with small variation. These results are also considered to be the reasons why AST and ALT were not related to Δglucose in the current study.

How about serum albumin, ChE, bilirubin, PT, and cholesterols? These parameters, reflecting the synthetic (albumin, ChE, prothrombin, and cholesterols) or elimination (bilirubin) functions of the liver [13], have been well entrenched in assessment of residual liver function. In particular, albumin, bilirubin, and PT constitute ‘Child-Pugh score,’ the indicator of both residual liver function and prognosis in the subjects with liver cirrhosis [13]. Although we speculate these parameters are associated with Δglucose during GST presumably reflecting HGO by glucagon, we failed to observe any relationship between them. Thus, we consider several possible reasons for our results. First, in the current study, we excluded the subjects with liver dysfunction or liver cirrhosis, who might be with impaired parameters such as albumin, bilirubin and PT. That is, similarly to AST and ALT, it can be mentioned that these parameters were also within a normal and a narrow range. Second, these parameters are influenced by various factors other than the synthetic and elimination functions in the liver. For instance, serum albumin levels are generally altered in the presence of proteinuria, hypermetabolism, and malnutrition. Additionally, serum bilirubin levels are also influenced by the existence of renal insufficiency and hemolysis [13]. We consider these variables are the indices not independently reflecting the residual liver function simply represented by synthetic and elimination functions in the liver, thus the results were away from our speculations.

Interestingly, we observed that Δglucose showed positive relationship with eGFR, and inverse relationship with serum creatinine and cystatin C. The kidney also plays critical roles in glucose metabolism via gluconeogenesis, glucose utilization, and glucose reabsorption. Renal glucose production is only through gluconeogenesis, since the kidney cannot produce glucose via glycogenolysis because the kidney has much smaller amount of glycogen than the liver and renal cells can synthesize glycogen but lack glucose-6-phosphatase [14]. Indeed, liver and kidney are almost equivalently involved in glucose production via gluconeogenesis in the post-absorptive state of normal subjects [4, 14]. Moreover, patients with T2DM exhibit abnormal increase of glucose release into the circulation through gluconeogenesis in both liver and kidney [5]. Thus, renal gluconeogenesis not only plays an important role in maintaining homeostasis, but also is involved in pathogenesis of T2DM. These previous findings and our observation provide us the speculation that Δglucose might reflect the capacity for glucose release via renal gluconeogenesis in T2DM.

However, the liver, but not the kidney, has been considered to be the main organ of gluconeogenesis regulated by glucagon. Previous study using isolated perfused rat kidney showed that glucagon does not stimulate gluconeogenesis in the kidney [15]. Furthermore, glucagon stimulates gluconeogenesis from glutamine and increases glucose output not in the kidney but in the liver in human [16]. On the other hand, a recent report revealed that both kidney and intestine induce gluconeogenesis by glucagon in a mouse model for liver-specific deletion of glucose production, concluding that current dogma concerning the roles of the liver and the extrahepatic gluconeogenic organs in glucose homeostasis needs to be revisited [17]. Therefore, we speculate that these observations might explain why glucagon-derived glucose increment was related to renal function in our current study.

Based on our results, we further hypothesized that Δglucose could be the determinant of the future renal function. Then, we performed multiple regression analysis to clarify the variables associated with eGFR after a year. Multiple regression analysis revealed that Δglucose was an independent determinant for the eGFR after 1 year, in addition to BMI, HbA1c, serum lipids, and UAE. Previous several studies already showed that obesity, glycemic control, serum lipids, and proteinuria were respectively the predictors for the development of chronic kidney disease [18, 19]. Our results are very interesting since it suggests that Δglucose is not only related to the present renal function but also a determinant for the future renal function in addition to other metabolic parameters which has been already examined. The correlation between Δglucose and renal function could be linked to the view that Δglucose is a proxy of the functional activity of the nephrons.

There are several limitations in this study. First, our current study was conducted with retrospective cross-sectional design. Second, our results were based on the relatively small population without common selection criteria at single hospital. Third, the subjects of the current study exhibited higher HbA1c levels among diabetic patients. Although we performed GST after adequate glycemic control, we need further investigation in populations with better glycemic control. Fourth, we did not investigate mechanistic analysis that can explain our results in this study, especially in the relationship between Δglucose and renal function. Prospective multicenter cohort studies with sufficient number of participants are necessary to clarify the relationship between Δglucose and various clinical parameters especially in renal functions for further understanding.

## Conclusions

In summary, this is the first report to show the clinical implication of Δglucose during GST. We observed the relationship between Δglucose and FFA. Furthermore, we revealed the relationship between Δglucose and both present and future renal function. Our present study suggests that Δglucose might be related to gluconeogenesis in the kidney, and we believe that Δglucose could be the determinant of future renal function in type 2 diabetes.

## Data Availability

The datasets used and/or analyzed during the current study are available from the corresponding author on reasonable request.

## Abbreviations

ALT: Alanine aminotransferase
AST: Aspartate aminotransferase
BMI: Body mass index
BW: Body weight
ChE: Cholinesterase
CPR: C-peptide immunoreactivity
DPP-4: Dipeptidyl peptidase-4
eGFR: Estimated glomerular filtration rate
FFA: Free fatty acids
GST: Glucagon stimulation test
HDL: High-density lipoprotein
HGO: Hepatic glucose output
HPLC: High performance liquid chromatography
IRI: Immunoreactive insulin
LDL: Low-density lipoprotein
PT: Prothrombin time
T2DM: Type 2 diabetes mellitus
UAE: Urinary albumin excretion
